# Effects of Interactive Video Game-Based Exercise on Balance in Diabetic Patients with Peripheral Neuropathy: An Open-Level, Crossover Pilot Study

**DOI:** 10.1155/2019/4540709

**Published:** 2019-03-06

**Authors:** Erica Shih-Wei Hung, Shih-Ching Chen, Fan-Chien Chang, Yaojung Shiao, Chih-Wei Peng, Chien-Hung Lai

**Affiliations:** ^1^Department of Physical Medicine and Rehabilitation, Taipei Medical University Hospital, Taipei, Taiwan; ^2^Department of Physical Medicine and Rehabilitation, School of Medicine, College of Medicine, Taipei Medical University, Taipei, Taiwan; ^3^Department of Vehicle Engineering, National Taipei University of Technology, Taipei, Taiwan; ^4^School of Biomedical Engineering, College of Biomedical Engineering, Taipei Medical University, Taipei, Taiwan; ^5^Graduate Institute of Biomedical Optomechatronics, College of Biomedical Engineering, Taipei Medical University, Taipei, Taiwan

## Abstract

*Purpose. *This study evaluated the effects of interactive video game-based (IVGB) exercise on balance in diabetic patients with peripheral neuropathy.* Materials and Methods. *Twenty-four patients were randomly assigned to two groups (12 participants per group). Group A received IVGB training for the first 6 weeks, with no exercise in the subsequent 6 weeks. Group B had no exercise for the first 6 weeks and then underwent IVGB training in the subsequent 6 weeks. For all participants, the Modified Falls Efficacy Scale (MFES), Time Up and Go (TUG) test, Berg Balance Scale (BBS), and Unipedal Stance Test (UST) were employed at weeks 0, 6, and 12 of the experiment.* Results.* BBS, right-leg UST, and TUG test scores significantly improved after IVGB intervention, whereas MFES and left-leg UST tended to improve after IVGB intervention.* Conclusions.* This study revealed that 6-week balance-based exercise training using the IVGB system exerted positive effects on functional balance in patients with diabetic peripheral neuropathy (DPN).

## 1. Introduction

Diabetes is a chronic metabolic disease that develops when either the pancreas does not produce enough insulin or body cells do not respond properly to the insulin produced. A WHO global report estimated that 422 million adults were diagnosed with diabetes in 2014, which is almost four times the number of cases diagnosed in 1980 [1]. Diabetes and its complications have a negative impact on patients' life quality and represent a major financial burden on national healthcare systems worldwide.

Diabetic peripheral neuropathy (DPN) is one of the most common complications of diabetes, and it results in impaired peripheral sensation, poor proprioception, slower walking speed, decreased step length [2], reduced ankle motion [3], declined muscle strength [4], and deficits in static and dynamic balance control [5, 6]. DPN affects nearly half of all individuals with diabetes, and the related functional changes significantly increase the risk of falls compared with healthy individuals of the same age [6]. Falls are often accompanied by traumatic injuries and fractures and are associated with nursing home and hospital admission costs, a decreased range of physical activity in daily life, and reduced self-efficacy as well as the psychological fear of falling again [7, 8]. The most consistent predictor of future falls is a diagnosis of gait or balance abnormalities [9]. Therefore, balance training exercises play a key role in reducing the frequency of falls and preventing further fall-related injuries [10].

Conventional balance exercise programs generally include static posture maintenance, standing activities, stepping, and treadmill walking. However, participation rates are low because these programs are often considered unattractive and boring due to repetitive nature of the exercises [11]. Fortunately, some studies have suggested that other types of balance training exercises such as Taichi softball and line dancing have positive effects on patients' balance, mobility, and quality of life [12, 13]. Although video games were originally designed for entertainment purposes, a variety of auditory and visual sensory feedback systems have been developed specifically for balance rehabilitation and training [14, 15]. Several recent studies have revealed that interactive video game-based (IVGB) exercises can ameliorate static and dynamic balance control in multiple sclerosis patients [16]; promote the correct weight shifting in stroke patients [17]; improve balance and attention deficits in chronic traumatic brain injury patients [18]; enhance functional balance and mobility in teenagers with cerebral palsy [19]. Some studies also suggested that IVGB exercise improve balance control, as well as self-reported balance confidence and mental health in healthy older population [11]. In one randomized controlled trial in 2013, Grewal et al. concluded that sensor-based interactive exercise with visual joint movement feedback could enhance postural stability in patients with DPN [20]. Moreover, compared with conventional therapy, balance training based on interactive video game programs is more enjoyable and attractive, and patients are thus more highly motivated to participate in such training activities, thereby increasing both the frequency of practice and the level of attention during training. Therefore, patients are more willing to maintain regular, long-term practice, and they demonstrate higher compliance [21].

Reducing the risk of falls and preventing their occurrence are essential for patients with DPN to remain functionally independent. However, little research on the use of IVGB therapy in patients with DPN has been conducted. Therefore, this study evaluated the effects of IVGB exercise on balance in diabetic patients with peripheral neuropathy. Both subjective and objective measures were used to determine whether IVGB exercise improves balance function.

## 2. Materials and Methods

### 2.1. Participants

Twenty-eight community-dwelling diabetic patients with peripheral neuropathy were recruited from the Outpatient Department of Taipei Medical University Hospital. The inclusion criteria were (1) 40–80 years of age; (2) medical diagnoses of diabetes under regular medication control and DPN confirmed using an electrodiagnostic test; (3) independent community ambulatory individuals; and (4) intact cognition (Minimental State Examination score of >24). Patients were excluded from this study if they had the following conditions: (1) other neurological diseases such as dementia, Parkinson's disease, spinal cord injury, or stroke; (2) severe visual impairment, musculoskeletal disorders, unhealed plantar ulceration, lower limb amputation, poor cardiopulmonary function, or other diseases affecting walking ability or any other disease due to which individuals were unable to walk without assistance; and (3) any other condition associated with a high risk of falling. Inability to follow simple instructions was another exclusion criterion.

**Figure 1:**
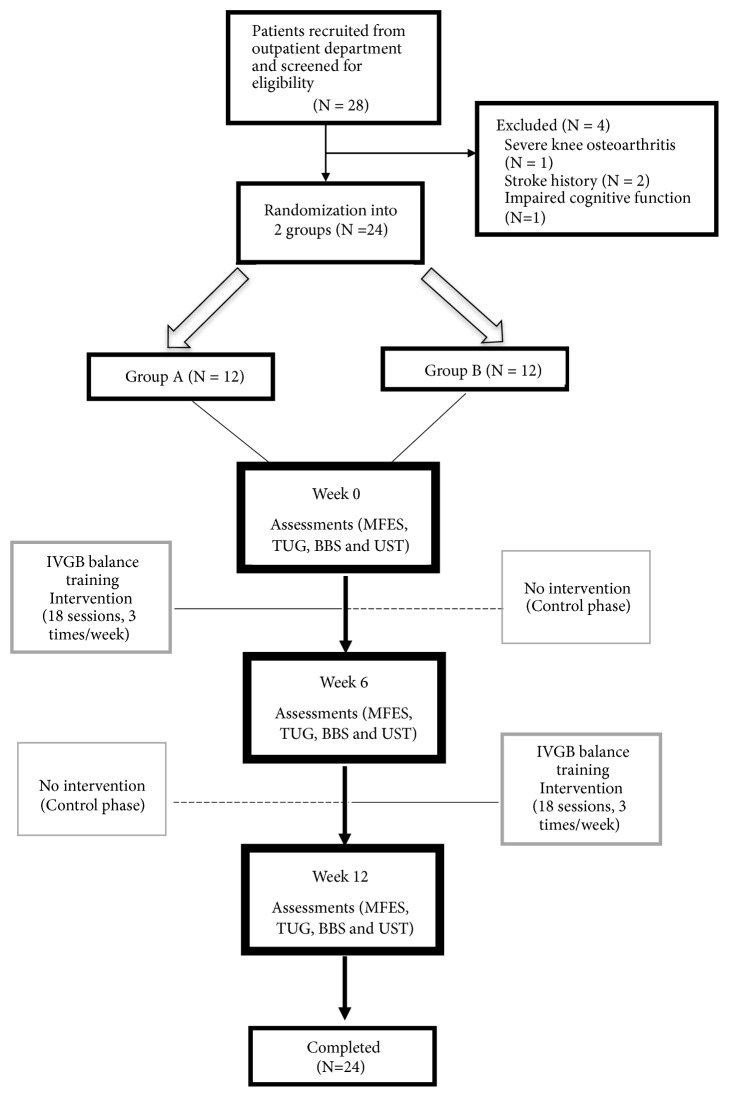
Flow chart of patient recruitment in this study.

After screening, four diabetic patients were excluded from the study, including one patient with bilateral severe knee osteoarthritis, two patients with a history of stroke, and one patient with impaired cognitive function. In total, 24 patients (8 men and 16 women) aged between 52 and 80 years (mean age = 66) were enrolled in this study (Figure 1). The subjects were randomized by block randomization with fixed block length of 6 and allocated into Groups A and B with 1:1 ratio. All participants were provided with a detailed explanation of the experimental protocol and provided signed informed consent before participating in the study. This study was conducted at Taipei Medical University Hospital and was approved by its Institutional Review Board with registration number TMU-JIRB 201308020. This experiment was performed in accordance with relevant guidelines and regulation at Taipei Medical University.

### 2.2. Study Design

Participants were randomly assigned to one of two groups, with 12 participants in Group A (2 men and 10 women, mean age = 71.0) as well as in Group B (6 men and 6 women, mean age = 66.5). Participants' demographic characteristics are summarized in Table 1. This study was a prospective, randomized, crossover, open-label 12-week trial. Group A received IVGB training for the first 6 weeks, with no exercise in the subsequent 6 weeks. Group B had no exercise in the first 6 weeks and then underwent IVGB training in the subsequent 6 weeks. For all participants, the IVGB balance training exercise program consisted of 30-minute sessions 3 times per week for 6 weeks. Outcomes were measured at weeks 0, 6, and 12. Our research assistant arranged for each participant to performs the tests and exercises. In addition, participants were not allowed to undergo any other physical (conventional) therapy related to lower limb strengthening and balance during the experimental period.

The IVGB balance training program employed a game console (XavixPORT, Shinsedai Company Limited), a 42-inch television, three software cartridges, a stepping mat, power cables connecting the console and television to a power source, and A/V cables connecting the console to the television.

### 2.3. Training Protocol

**Figure 2:**
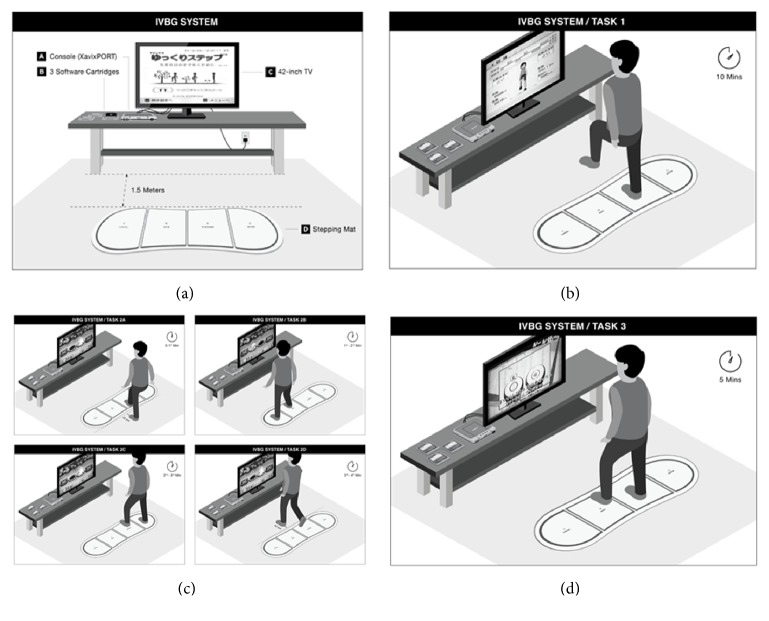
Protocol of IVGB intervention. (a) IVGB system set-up. (b) Task 1: stepping exercise. (c) Task 2: hamsters game. (d) Task 3: drumming game.

Participants in Group A received IVGB intervention for the first 6 weeks (intervention phase), with no exercise in the subsequent 6 weeks (control phase), whereas participants in Group B received no IVGB intervention in the first 6 weeks (control phase), followed by 6 weeks of IVGB intervention (intervention phase). The IVGB intervention protocol consisted of 30-minute training sessions comprising four tasks designed to focus on lower limb strength, balance, and coordination training. As shown in Figure 2(a), throughout the experiment, participants performed exercise on the IVGB system in a quiet room, and the stepping mat was placed 1.5 m in front of the television. In task 1, participants mimicked the movements of the on-screen virtual character by stepping on the corresponding place on the mat with sensory detection. Participants performed 10-minute stepping exercise, in which they raised each knee to waist level for consistent measurement (Figure 2(b)). During the exercise, participants kept their trunk in a steady and upright position to minimize compensation and deviation. In task 2, as shown in Figure 2(c), participants played a 5-minute stepping hamsters game, in which they stood 12 cm behind the mat as the starting position. In the first minute, participants stepped forward with one leg to strike the hamsters emerging from holes displayed on the television screen (Figure 2(c)). In the second minute of the game, participants stood on the left end of the mat and stepped laterally with their right leg to strike the hamsters. In the third minute, they stood on the right end of the mat and stepped laterally with their left leg to strike the hamsters (Figure 2(c)). In the fourth minute of the game, participants stood 12 cm in front of the mat and stepped backward with one leg to strike the hamsters (Figure 2(c)), and in the fifth minute they repeated the session in which they had the lowest score compared with previous sessions. In task 3, participants engaged in 5-minute drumming activity by following the rhythm of two slow and two fast songs and stepping on the corresponding spots on the mat, as per the game instructions (Figure 2(d)). In task 4, participants repeated the same 10-minute stepping exercise as in task 1. All training courses were carried out by the research assistant, a well trained and certified physical therapist.

In summary, the frequency, intensity, time, and type (FITT) programming guidelines for IVGB exercise intervention in the present study were defined as follows: (1) frequency, 3 times per week for 6 weeks; (2) intensity, 30-minute session composed of 4 training tasks; (3) time, 30 minutes; and (4) type, strength, balance and coordination training

### 2.4. Outcome Measurements

Balance assessments consisted of both subjective and objective measures, including the Modified Falls Efficacy Scale (MFES), Timed Up and Go (TUG) test, Berg Balance Scale (BBS), and Unipedal Stance Test (UST). For all participants, these tests were conducted at weeks 0, 6, and 12 of the experiment. All assessments were carefully designed and performed by the same senior resident from Department of Physical Medicine of Rehabilitation at Taipei Medical University Hospital.

### 2.5. Modified Falls Efficacy Scale (MFES)

MFES is a 14-item questionnaire related to daily indoor and outdoor physical activities, and it represents an expanded version of the original 10-item Falls Efficacy Scale. It is a reliable and valid measure of falls self-efficacy [22]. It is a 10-point visual analog scale of confidence level in completing a particular activity (item) without falling, rated from 0 to 10, where 0 denotes not confident or sure at all, and 10 denotes completely confident or sure. The total score for the 14 items ranges from 0 to 140.

### 2.6. Timed Up and Go (TUG) Test

The TUG test is a quick, reliable, valid, and accurate method commonly used to test dynamic stability and functional mobility [23–25]. In the test, participants stand up from a 46-cm-high armchair with back support, walk straight for 3 m, turn around, walk back to the chair, and sit down as quickly and safely as possible. The timing starts when the investigator says “go” and stops when the participant sits back down on the chair. In our study, each participant had three chances to complete the TUG test in each session, and the best result was recorded for each participant.

### 2.7. Berg Balance Scale (BBS)

BBS is a performance-oriented measurement of an individual's static and dynamic balance in daily life activities, and it may be easily performed in a rehabilitation setting or an outpatient clinic [4, 5]. It consists of 14 functional tasks of varying difficulty, including sitting, standing, changing posture, transfers, reaching forward, retrieving objects, turning, tandem stance, and one-leg stance. It is a valid tool used in both clinical practice and research to evaluate the efficacy of intervention and provide a quantitative description of balance function [26, 27]. The ability to perform each a task is scored on a scale of 0–4, ranging from inability to independently perform the task to successfully completing it, respectively. The maximum possible score for the 14 functional tasks is 56.

### 2.8. Unipedal Stance Test (UST)

UST is a simple and reliable method of testing static balance as well as a strong predictor of falls [28, 29]. In our study, participants raised one leg to the ankle level or higher without touching the other leg or using any assistance and then stood on that leg for as long as possible barefoot and with eyes open. The observer measured the length of time for which participants maintained balance; that is, until they were no longer able to keep the leg raised at or above the ankle level or when both legs touched the ground. The observer stopped counting at 45 seconds, recording this as the time for any participant who maintained balance for a longer time. Each participant performed the test three times for both right and left legs, with the best result for each leg recorded [30].

### 2.9. Statistical Analysis

All data are expressed as mean ± standard error. The chi-squared test was used to identify significant differences in the general characteristics of these two random samples. We used nonparametric statistics, namely a Friedman two-way analysis of variance (ANOVA) test, to compare distributions of all outcome measurements. All significant values derived from the Friedman test were further analyzed in a post hoc test. The level of significance was set at P < 0.05. The difference in the outcome variables between intervention and nonintervention was analyzed with a general linear mixed model. An estimation of fixed effects was applied to treatment and period effects where subject within each sequence was treated as random effects. This linear mixed model detected a period effect and a significant association between the intervention and responses. In addition, Mann–Whitney U test was used to compare changes in variable outcomes of the IVGB intervention phase with changes in variable outcomes of the control phase. All statistical calculations were performed using SPSS 18.0 (SPSS, Chicago, IL, USA).

## 3. Results

### 3.1. Effect of Intervention

#### 3.1.1. Group A

Scores of BBS and UST performed under the eyes open condition improved significantly after IVGB balance exercise (0–6 weeks). The mean BBS score at the week-6 assessment was 52.33 ± 3.77 and increased in comparison with the score of 48.92 ± 4.81 at week 0 (P = 0.009). BBS scores decreased slightly to 50.17 ± 4 from weeks 6 to 12 (control phase), but this difference was not significant. Overall, from weeks 0 to 12, the BBS score improved from 48.92 ± 4.81 (week 0) to 50.17 ± 7.37 (week 12). After 6 weeks of intervention, the mean best results for the right-leg UST score also improved significantly from 10.52 ± 10.19 seconds at week 0 to 21.68 ± 17.45 seconds at week 6 (P = 0.029). The right-leg UST score decreased slightly to 18.89 ± 14.71 seconds from weeks 6 to 12 (control phase), but this difference was not significant. After 6 weeks of IVGB intervention, the mean best results for the left-leg UST score also improved significantly from 10.05 ± 8.71 seconds at week 0 to 22.71 ± 18.18 seconds at week 12 (P = 0.008). Moreover, participants showed increased MFES score and a shorter task completion time in the TUG test after 6 weeks of IVGB intervention (Table 2).

#### 3.1.2. Group B

BBS and UST scores under the eyes open condition improved significantly after IVGB balance exercise intervention. The mean BBS score at the week-12 assessment was 50.92 ± 5.57, which was significantly higher than the score of 45.67 ± 8.91 initially assessed at week 6 (P < 0.001). The mean best result for the right-leg UST was 21.66 ± 13.86 seconds at week 12 compared with 15.30 ± 15.68 seconds at week 0 (P = 0.028). The mean best result for the left-leg UST was 22.89 ± 17.09 seconds at week 12 compared with 16.60 ± 17.15 seconds at week 0 (P = 0.037). Moreover, after 6 weeks of IVGB intervention, the MFES score exhibited an increasing trend, and in the TUG test, participants demonstrated a shorter task completion time (Table 2).

**Figure 3:**
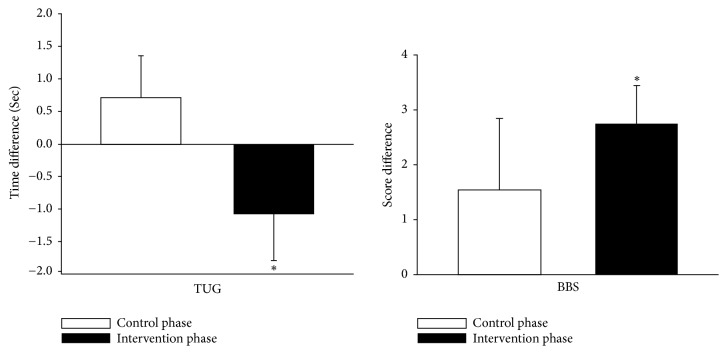
Combined changes in variable outcomes of the intervention phase in Groups A and B versus combined changes in variable outcomes of the control phase in Groups A and B. TUG test, Timed Up and Go test. BBS, Berg Balance Scale. The time/score differences for TUG test and BBS in the intervention phase were calculated by subtracting the scores/time for week 6 from those for week 0 in Group A and subtracting the scores/time for week 12 from those for week 6 in Group B, whereas their time/score differences in the control phase were calculated by subtracting the scores/time for week 12 from those for week 6 in Group A and subtracting the scores/time for week 6 from those for week 0 in Group B. *∗* P < 0.05, difference between mean increase in participants with intervention and those without intervention as determined by the Mann–Whitney U test.

In addition, when we summed up all changes in variable outcomes of the intervention phase from Groups A and B and compared them with changes in variable outcomes of the control phase from these groups, significant improvements in the TUG test (P = 0.013) and BBS (P = 0.004) scores were observed (Figure 3). These results were confirmed by the general linear mixed model.

## 4. Discussion

This study demonstrated that 6-week balance-based exercise training using the IVGB system had positive effects on functional balance in patients with DPN. BBS, UTS, and TUG test scores significantly improved after intervention.

BBS is a comprehensive performance-oriented assessment of functional balance that mimics daily life activities and is considered a crucial predictor of falls [31]. Previous studies recommended that there was relevance between BBS scores and the increase of fall risk. Lajoie and Gallagher showed that the sensitivity and specificity of correctly classifying fallers and nonfallers with a cut-off value of 46 was 82.5% and 93%, respectively [32]. Shumway-Cook et al. stated that declining of BBS scores was related to increasing fall risk. For scores between 56 and 54, every 1-point drop in BBS referred to an increase of 3% to 4% risk of falling. For scores between 54 and 46, every 1-point drop referred to an increase of 6% to 8% risk of falling. Any score below 36 was associated with almost 100% risk of falling [33]. Therefore, a change of 1 point could bring out very different predicted probability for a fall. In this study, the BBS score significantly improved after IVGB intervention in both Groups A and B. In Group A, BBS scores improved from 48.92 ± 4.81 (before intervention) to 52.33 ± 3.77 (after intervention). In Group B, BBS scores increased from 45.67 ± 8.91 (before training) to 50.92 ± 5.57 (after training). These results implied that risk of falling in persons with DPN might decrease after IVGB training in present study. However, it is still necessary to follow the long-term falling rate after IVGB training in patients with DPN in future study. BBS scores in Group B increased significantly after 6 weeks of IVGB intervention. Moreover, BBS scores after the intervention phase were significantly improved compared with those recorded after the control phase while we combined outcome measurements in Groups A and B.

In Group A, the maximum time of right-leg UST significantly increased after IVGB intervention. However, although significant improvement in left-leg UST was observed from weeks 0 to 12, an increasing trend in left-leg UST increase was only observed from weeks 0 to 6 (i.e., the intervention phase). Notably, no significant differences in right-leg UST measurements were recorded between weeks 6 and 12 (i.e., the intervention phase) in Group B, whereas a significant improvement was observed from weeks 0 to 6, when no intervention took place. Similar outcomes were observed for left-leg UST in Group B, where a significant difference was observed in the control phase, rather than in the intervention phase. This observation may be attributed to the active education provided to participants at the beginning of the study, including information on the consequences of DPN and the necessity of a regular exercise routine—especially balance training—in their daily lives. It is therefore likely that the knowledge that participants received at the beginning of the study prompted them to include balance exercises in their daily routine and prompted them to become more aware of their posture stability, which facilitated improvement of UST even in the control phase. Nevertheless, an overall significant improvement of UST was observed from weeks 0 to 12 in Group B.

MFES is a self-administered questionnaire that assesses an individual's level of confidence in performing daily activities without falling. In this study, in Groups A and B, the MFES score improved after intervention from 115.25 to 127.83 and from 108.5 to 114.75, respectively. This suggests that after intervention, participants in both groups were more confident in performing daily activities without the fear of falling.

In Groups A and B, the time required to complete the TUG test showed a decreasing trend after 6 weeks of intervention, from 9.58 to 9.35 seconds and from 11.47 to 9.55 seconds, respectively. Moreover, in the TUG test, the completion time in the combined intervention phase (i.e., Groups A and B) was significantly shorter than that in the combined control phase (P = 0.013).

Many studies have supported the efficacy of strength and balance training for patients with DPN and have indicated a significant improvement in balance and muscle strength, increased walking speed, and reduced fear of falling with fewer incidents of falls [34, 35]. Our intervention focused on dynamic tasks consisting of lower limb strength training, reactive balance control, and environmental interaction. Stepping exercises enhance lower limb strength and trunk control. Stepping on targets in different directions requires left/right and forward/backward weight shifting, thus posing a challenge to dynamic balance and functional mobility. Grewal et al. suggested that interactive balance training with visual joint movement feedback improves postural stability in patients with DPN [36]. Drumming games reinforce training of coordination of lower extremities, trunk control, and single leg support. Several studies have indicated the positive effects of lower limb strengthening and dynamic balance training on gait endurance, postural stability, and fall prevention [37, 38]. The IVGB system used in this study provided participants with an interactive environment assisted by instant visual and auditory feedback, including on-screen display of the lifting time for each leg during the stepping exercise and the scores for the stepping hamsters and drumming games. Participants were thus made aware of their postural stability and coordination during exercise and could compare their performance with scores of previous sessions.

The main limitation of this study was its small-sample size, although a crossover design was used to minimize the effect of small-sample statistics. The target population was diabetic patients with peripheral neuropathy. However, many such patients refused to join the study for several reasons. First, some patients did not feel the need for balance training because they could still walk independently. Second, many patients thought it inconvenient to come to the hospital three times a week to participate in this study. In the future, we hope to include IVGB training in home-based rehabilitation programs to make it more accessible and easier to use. Moreover, patients should be educated about the necessity of balance exercise in their daily lives. The lack of a washout phase was another limitation in current study. The washout phase can be a good choice in crossover study. Nevertheless, present study did not design a washout period because of the following reasons. One of the reasons was that we assumed current study might decrease higher-order carryover effects because the second phase is no intervention for six weeks. Previous study suggested that a washout period was often unnecessary for diabetes studies if the second period is long enough to diminish carryover effect [39]. Another reason was that we would like to reduce dropouts and missing data that usually make a larger bias on crossover trials than on parallel group trials [40, 41]. However, the difference in the scores of BBS and TUG between intervention and nonintervention was significant while used a mixed model for fixed effects to examine the treatment and period effects. In this study, the comparison between the effect of IVGB balance training and conventional physical therapy was not investigated. Many studies have indicated that strength and balance training is safe and feasible for diabetic patients with peripheral neuropathy and can significantly reduce fall risks [34]. Future research with a more rigorous design to compare the effect of IVGB training with that of conventional physical therapy is recommended. In addition, the total length of our experiment was 12 weeks, with an intervention period and follow-up period of 6 weeks each. Further research with a longer trial and follow-up period is necessary to augment the intervention effect and monitor its retention.

## 5. Conclusions

This study revealed that 6-week balance-based exercise training using the IVGB system had positive effects on functional balance in patients with DPN. BBS, UTS, and TUG test scores significantly improved after intervention.

## Figures and Tables

**Table 1:** Demographic characteristics of participants.

	Group A (N=12)	Group B (N=12)	*P *value
Age (years)	71.0 (1.22)	66.5 (2.10)	0.337
Gender (Male/ Female)	2/10	6/6	0.178
Height (cm)	155.14 (2.02)	165.58 (2.72)	0.571
Weight (kg)	60.04 (3.92)	71.00 (2.84)	0.500
BMI	24.76 (1.26)	25.83 (0.72)	0.404
MMSE	27.58 (0.59)	27.75 (0.69)	0.381
Regular exercisers (three times/week)	3	5	0.386
Number of falls in the past year	5	3	0.386

Data are presented as mean (standard error) or numbers.

Group A: Intervention 6 weeks (three times/week) after assessment, then control 6 weeks (three times/week).

Group B: Control 6 weeks (three times/week) after assessment, then intervention 6 weeks (three times/week).

BMI, body mass index

MMSE, Mini-Mental State Examination

**Table 2:** Outcome variables of the baseline, 6th week, and 12th week.

Group	A				B			
	Baseline	6th week	12th week	*P *value	Baseline	6th week	12th week	*P *value
MFES (score)	115.25 (9.06)	127.83 (4.58)	125.83 (6.36)	0.046*∗*	103.83 (12.61)	108.58 (11.54)	114.75 (9.81)	0.109
TUG (second)	9.58 (0.87)	9.35 (1.09)	9.58 (1.22)	0.089	10.30 (1.09)	11.47 (1.87)	9.55 (1.35)	0.205
BBS (score)	48.92 (1.29)	52.33 (1.01)^#^	50.17 (1.97)	0.009*∗*	43.58 (2.87)	45.67 (2.69)	50.92 (1.68)^**$,§**^	0*∗*
UST (second)								
R.EO-Max.	10.52 (3.07)	21.68 (5.26)^#^	18.89 (3.93)^**§**^	0.029*∗*	15.30 (4.73)	21.60 (4.87)	21.66 (4.18)^**§**^	0.028*∗*
L.EO-Max.	10.05 (2.18)	16.28 (4.55)	22.71 (4.86)^**$,§**^	0.008*∗*	16.60 (5.17)	19.60 (5.28)	22.89 (5.15)^**§**^	0.037*∗*

Data are presented as mean (standard error) or numbers.

MFES, Modified Falls Efficacy Scale

TUG test, Timed Up and Go test

BBS, Berg Balance Scale

R.EO-Max., Unipedal Stance Test with eyes open, right leg

L.EO-Max., Unipedal Stance Test with eyes open, left leg

^*∗*^P < 0.05, determined by Friedman two-way ANOVA

^#^P < 0.05, difference between outcome variables at baseline and week 6 as determined by post hoc test

^**$**^P < 0.05, difference between outcome variables at weeks 6 and 12 as determined by post hoc test

^**§**^P < 0.05, difference between outcome variables at baseline and week 12 as determined by post hoc test

## Data Availability

The real-valued datasets used to support the findings of this study have been deposited in the ClinicalTrials.gov repository (ID: NCT03676595).

## Supplementary Materials

Supplementary Materials The TIDierR (Template for Intervention Description and Replication) checklist was included as a supplementary file along with this manuscript.
